# Influence of green tea consumption on endoxifen steady-state concentration in breast cancer patients treated with tamoxifen

**DOI:** 10.1007/s10549-020-05829-6

**Published:** 2020-08-16

**Authors:** C. Louwrens Braal, Koen G. A. M. Hussaarts, Lieke Seuren, Esther Oomen-de Hoop, Peter de Bruijn, Stefan A. J. Buck, Monique E. M. M. Bos, Martine F. Thijs-Visser, Hanneke J. M. Zuetenhorst, Daniëlle Mathijssen-van Stein, Mijntje B. Vastbinder, Roelof W. F. van Leeuwen, Teun van Gelder, Stijn L. W. Koolen, Agnes Jager, Ron H. J. Mathijssen

**Affiliations:** 1 Department of Medical Oncology, Erasmus MC Cancer Institute, Dr. Molewaterplein 40, CN, PO Box 2040, 3015 Rotterdam, The Netherlands; 2 Department of Medical Oncology, Ikazia Hospital, Rotterdam, The Netherlands; 3 Department of Internal Medicine, Franciscus Gasthuis & Vlietland, Schiedam, The Netherlands; 4 Department of Internal Medicine, IJsselland Hospital, Capelle aan den IJssel, The Netherlands; 5 Department of Hospital Pharmacy, Erasmus University Medical Center, Rotterdam, The Netherlands; 6 Department of Clinical Pharmacy and Toxicology, Leiden University Medical Center, Leiden, The Netherlands

**Keywords:** Tamoxifen, Green tea extract, Epigallocatechin-3-gallate (EGCG), Herb–drug interaction, Pharmacokinetics, Toxicities, Breast cancer

## Abstract

**Background:**

Many cancer patients use additional herbs or supplements in combination with their anti-cancer therapy. Green tea—active ingredient epigallocatechin-3-gallate (EGCG)—is one of the most commonly used dietary supplements among breast cancer patients. EGCG may alter the metabolism of tamoxifen. Therefore, the aim of this study was to investigate the influence of green tea supplements on the pharmacokinetics of endoxifen; the most relevant active metabolite of tamoxifen.

**Methods:**

In this single-center, randomized cross-over trial, effects of green tea capsules on endoxifen levels were evaluated. Patients treated with tamoxifen for at least 3 months were eligible for this study. After inclusion, patients were consecutively treated with tamoxifen monotherapy for 28 days and in combination with green tea supplements (1 g twice daily; containing 300 mg EGCG) for 14 days (or vice versa). Blood samples were collected on the last day of monotherapy or combination therapy. Area under the curve (AUC_0–24h_), maximum concentration (*C*_max_) and minimum concentration (*C*_trough_) were obtained from individual plasma concentration–time curves.

**Results:**

No difference was found in geometric mean endoxifen AUC_0–24h_ in the period with green tea versus tamoxifen monotherapy (− 0.4%; 95% CI − 8.6 to 8.5%; *p* = 0.92). Furthermore, no differences in *C*_max_ (− 2.8%; − 10.6 to 5.6%; *p* = 0.47) nor *C*_trough_ (1.2%; − 7.3 to 10.5%; *p* = 0.77) were found. Moreover, no severe toxicity was reported during the whole study period.

**Conclusions:**

This study demonstrated the absence of a pharmacokinetic interaction between green tea supplements and tamoxifen. Therefore, the use of green tea by patients with tamoxifen does not have to be discouraged.

## Introduction

Breast cancer is the most commonly diagnosed type of cancer among women [1]. In the adjuvant treatment of hormone sensitive breast cancer, tamoxifen is the most frequently used and an effective oral endocrine therapy [2]. Many cancer patients—with estimates up to 80%—use complementary and alternative medicines in combination with their anti-cancer therapy [3–7]. One of the most popular herbal supplements among breast cancer patients are green tea (*Camellia sinensis*) supplements [4, 5, 8].

Green tea contains a large number of bioactive compounds, such as catechins and flavonoids [9, 10]. The active pharmacological ingredient of green tea is epigallocatechin-3-gallate, EGCG [11]. EGCG is believed to contribute to various cancer-preventive effects resulting from its high antioxidant potential [11–14]. In vitro and animal studies reported a number of cancer-preventative effects of EGCG including: attenuation of oxidative stress, inhibition of angiogenesis, induction of apoptosis and alterations in expression of cell cycle regulatory proteins [11, 12, 14–17]. None of these effects have been proven clinically. However, there are also signs that green tea and associated substances can influence other prescribed drugs. For example, it has been reported that EGCG could significantly reduce the systemic exposure of nadolol, folic acid and digoxin in subjects with approximately 85%, 39% and 31%, respectively [18–20]. Moreover, EGCG significantly increased the bioavailability of for example simvastatin and verapamil in rat studies [21, 22]. The described interactions with these drugs are the result of altered bioavailability or decreased metabolism, and can mechanistically be explained by inhibition of influx transporter organic anion transporter polypeptide (OATP) or efflux transporter P-glycoprotein and several phase I and II metabolizing enzymes (e.g., cytochrome P450 (CYP) 3A and UDP-glucuronosyltransferase (UGT)) [18–27]. Simultaneous administration with green tea is therefore not recommended for these drugs. However, the impact of green tea on tamoxifen pharmacokinetics remains unclear.

**Fig. 1:**
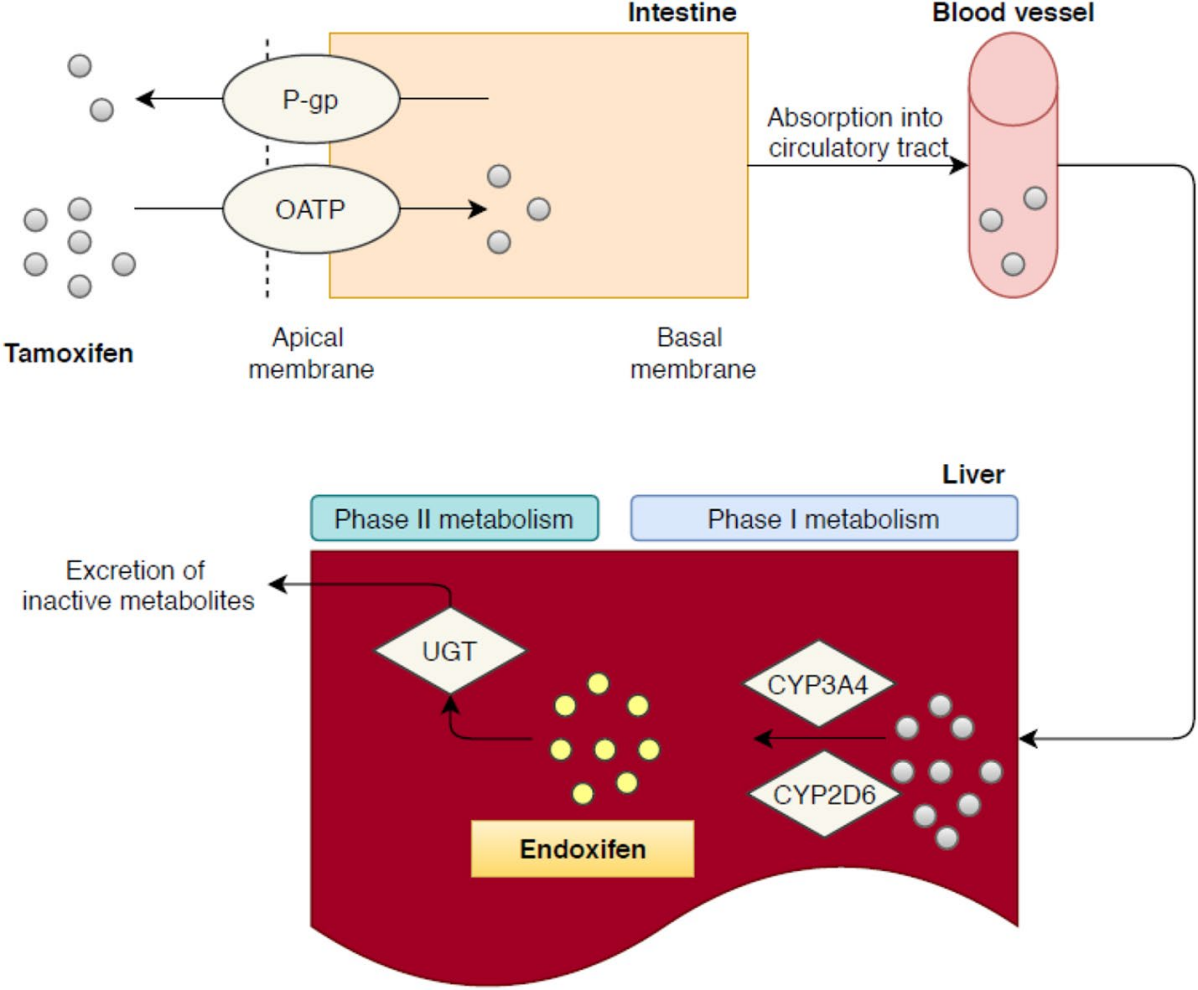
Main metabolism pathway of tamoxifen. After absorption tamoxifen is metabolized mainly by CYP2D6 in its active metabolite endoxifen. Tamoxifen relies on phase II metabolism before it can be excreted from the body. Endoxifen is ultimately glucuronidated into endoxifen-glucuronide mainly by UGTs. Several in vitro studies suggest inhibition by green tea of several phase I enzymes (CYP2D6 and CYP3A4) and inhibition of several drug-transporters which the efflux transporter P-gP (ABCB1) and sever influx-transporters like OATP. *P-gP* P-glycoprotein, *CYP* cytochrome P450, *OATP* organic anion transporting polypeptide, *UGT* UDP-glucuronosyltransferase

Tamoxifen pharmacokinetics depends on a multi-pathway biotransformation (Fig. 1) [28]. After hepatic uptake by—among others—OATP1B1, the cytochrome P450 iso-enzymes CYP2D6 and CYP3A4 metabolize tamoxifen into the main metabolite endoxifen [28–31]. Endoxifen is ultimately glucuronidated by UGT into an inactive metabolite and excreted through bile and feces [30]. In view of the involvement of drug transporting proteins and metabolizing enzymes, green tea could potentially interfere with the tamoxifen metabolism. Herb–drug interactions with tamoxifen could negatively impact the pharmacokinetic profile, as was previously shown with the combination of tamoxifen and curcumin [32]. Therefore, the primary objective of this study was to evaluate the possible pharmacokinetic interaction between green tea supplements and tamoxifen. The secondary objective was to assess the safety profile of green tea in combination with tamoxifen.

## Methods

### Study design

This single-center, randomized, two-armed, open-label, pharmacokinetic cross-over trial aimed to investigate the endoxifen exposure in breast cancer participants using tamoxifen with or without green tea. The study protocol was written in conformity with the Declaration of Helsinki and approved by the Local Medical Ethics Committee and registered at the Netherlands Trial Registry (Number NL8144). Enrollment took place after written informed consent at the Erasmus University Medical Center, Rotterdam, The Netherlands. Patients with a confirmed histological or cytological diagnosis of primary breast cancer, a World Health Organization (WHO) performance status of ≤ 1 and on tamoxifen treatment at a stable dose of 20 or 40 mg q.d. for at least 3 months (ensuring steady-state concentration) were included. Participant demographics, medical history, CYP2D6 phenotype status and serum biochemistry were assessed before study entry. Participants were excluded if they were CYP2D6 poor or ultra-rapid metabolizers or if they had an impaired drug absorption. Furthermore, all participants were required to abstain from herbal or dietary supplements and strong inhibitors or inducers of CYP3A4, CYP2D6, UGT and P-glycoprotein. Depending on randomization, participants either started with tamoxifen monotherapy (20 or 40 mg q.d.; 10 AM) for 28 consecutive days or tamoxifen and green tea (1000 mg b.i.d.; containing 150 mg of EGCG; 10 AM and 10 PM) concomitantly for 14 consecutive days. This dose of green tea capsules is equivalent to approximately 5 to 6 cups of regular green tea and is also in line with previous clinical studies. Thereafter, participants received tamoxifen and green tea concomitantly for 14 consecutive days or tamoxifen monotherapy for 28 days, respectively. The green tea capsules were manufactured by a qualified Dutch Pharmacy (NatuurApotheek, Pijnacker, the Netherlands) and the batch was provided with a certificate of analysis for verification of the EGCG content. Participants were hospitalized for 24-h pharmacokinetic blood sampling on days 14 and 42, after one night of fasting. Blood samples were collected periodically at 13 predefined time points (*t* = 0, 0.5, 1.5, 2, 2.5, 3, 3.5, 4, 6, 8, 12 and 24 h after tamoxifen intake) and after processing to plasma stored at − 80 °C until analysis. Plasma samples were analyzed by a validated liquid chromatography–tandem mass spectrometry (UPLC–MS/MS) method in accordance with U.S. Food and Drug Administration (FDA) bioanalytical method validation guidelines [33]. Adverse events were graded using the Common Terminology Criteria for Adverse Events version 5.0 (CTCAEv.5, National Cancer Institute, Bethesda, MD, USA).

### Pharmacokinetic analysis

A non-compartmental pharmacokinetic analysis of steady-state concentrations was performed using Phoenix WinNonlin version 8.1 (Pharsight, a Certara Company, Princeton, NJ, USA). Main pharmacokinetic parameters including area under the curve (AUC_0–24h_), maximum observed concentration (*C*_max_) and minimum observed concentration (*C*_trough_) were constructed by individual plasma concentration–time curves.

### Statistical analysis

The main objective of this trial was to compare the concentration of endoxifen with and without green tea supplements by comparing the AUC_0–24h_ between days 14 and 42, where one comparison was made: endoxifen monotherapy versus combined with green tea supplements. A relative difference in AUC_0–24h_ of at least 25% was considered to be clinically relevant and the within-patient deviation was assumed to be 20%. Given a power of 90% and a two-sided *α* of 5%, this resulted in a sample size of 14 evaluable patients (7 in both treatment arms). Analyses of AUC of tamoxifen, and *C*_trough_ and *C*_max_ of both endoxifen and tamoxifen were performed on log-transformed observations since these are assumed to follow a log-normal distribution. Estimates for the mean differences in *C*_trough_ and *C*_max_ were obtained for one comparison (tamoxifen concomitantly with green tea monotherapy versus tamoxifen monotherapy) separately using a linear mixed effect model treatment with sequence, and period as fixed effects and subject within sequence as a random effect. Variance components were estimated based on restricted maximum likelihood (REML) methods, and the Kenward–Roger method of computing the denominator degrees of freedom was used. The antilog were taken from the effect estimate and corresponding 95% confidence interval boundaries for the comparisons of tamoxifen concomitantly with green tea versus tamoxifen monotherapy to interpret the results (interpreted as ratios of the geometric means).

## Results

### Trial participants

Between October 2019 and February 2020, a total of 14 breast cancer patients were enrolled. All participants completed this trial and were evaluable. An overview of baseline characteristics is presented in Table 1. Participants were predominantly of Caucasian origin (86%) and were extensive metabolizers of CYP2D6 (79%). All participants were treated with adjuvant tamoxifen in this trial. The vast majority of patients used tamoxifen in a dose of 20 mg once daily (93%) and 1 patient used tamoxifen in a dose of 40 mg once daily (7%). In addition, the median duration of tamoxifen use before enrollment in this trial was 11.8 (range 6.0 to 12.9) months.

**Table 1:** Baseline characteristics of evaluable participants (*N* = 14)

Characteristic	*N* (%) or median (range)
**Sex**	
Female	14 (100%)
Male	0 (0%)
**Age (years)**	58.5 (50.8–68.3)
**BMI (kg/m**^**2**^**)**	27.4 (23.9–28.5)
**WHO performance status**	
0	12 (86%)
1	2 (14%)
**Ethnic origin**	
Caucasian	12 (86%)
Afro-Caribbean	2 (14%)
**CYP2D6 phenotype**	
EM	11 (79%)
IM	3 (21%)
**Biochemistry**	
AST (U/L)	21 (17.8–27.0)
ALT (U/L)	15 (11.8–21.0)
ALP (U/L)	53.5 (43–67)
GGT (U/L)	21 (16.5–29.5)
Total bilirubin (µmol/L)	6 (5.3–8.5)
Albumin (g/L)	36 (35–37)
LD (U/L)	189 (181.5–196.5)
Hb (mmol/L)	8.1 (7.7–8.3)
Creatinine (µmol/L)	76.5 (71.8–87.3)
**Previous treatment**	
Surgery	14 (100%)
Radiotherapy	9 (64%)
Chemotherapy	3 (21%)
**Tamoxifen dose**	
20 mg	13 (93%)
40 mg	1 (7%)
Duration of adjuvant tamoxifen use (months)	11.8 (6.0–12.9)

*BMI* body mass index, *EM* extensive metabolism, *IM* intermediate metabolism, *AST* aspartate aminotransferase, *ALT* alanine aminotransferase, *ALP* alkaline phosphatase, *GGT* gamma-glutamyltransferase, *LD* lactate dehydrogenase, *Hb* hemoglobin

### Pharmacokinetics

**Fig. 2:**
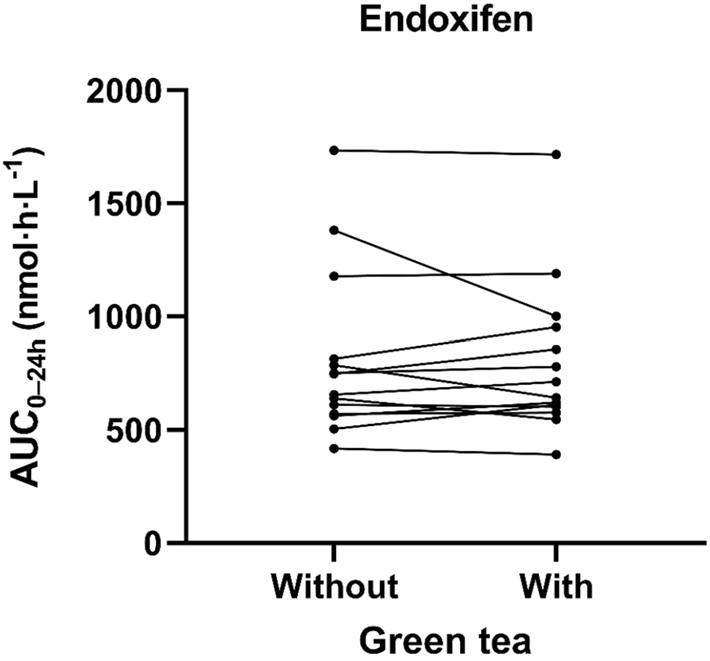
Pharmacokinetics of endoxifen without and with concomitant green tea supplements

**Fig. 3:**
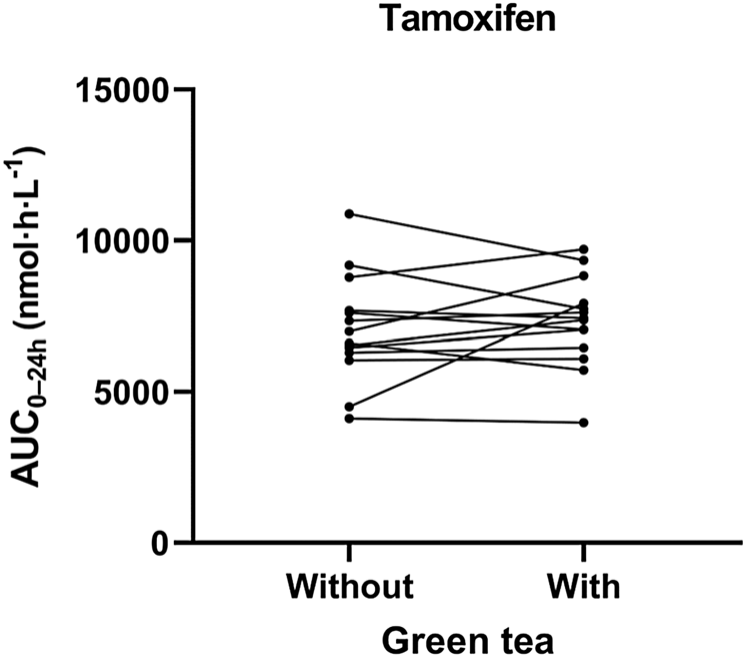
Pharmacokinetics of tamoxifen without and with concomitant green tea supplements

Tamoxifen and endoxifen levels were detectable in all collected blood samples. Estimates of main pharmacokinetic parameters for tamoxifen monotherapy versus tamoxifen with green tea supplements are presented in Table 2. The individual AUC values for endoxifen and tamoxifen exposure without and with green tea supplements are displayed in Figs. 2 and 3. The geometric mean of endoxifen AUC_0–24h_ during concomitant administration of green tea was comparable to tamoxifen monotherapy [746 nmol h/L; coefficient of variation (CV): 38.6% vs 749 nmol h/L; CV 41.1%]. The corresponding relative difference (RD) in endoxifen AUC_0–24h_ between the cycle with and without green tea was − 0.4% (95% CI − 8.6 to 8.5%; *p* = 0.92). Endoxifen geometric means of *C*_max_ 38.5 nmol/L; CV 37.3% vs 39.6 nmol/L; CV 41.7% and *C*_trough_ 32.2 nmol/L; CV 34.1% vs 31.9 nmol/L; CV 39.8% also did not significantly differ between with or without green tea.

**Table 2:** Main pharmacokinetic parameters of tamoxifen and endoxifen

PK parameters	Tamoxifen monotherapy^a^	Tamoxifen with green tea^a^	*p*-value	Relative difference (%) (95% CI)
**Endoxifen**				
AUC_0–24h_ (nmol·h/L)	749 (41.1)	746 (38.6)	0.92	− 0.4 (− 8.6 to 8.5)
* C*_max_ (nmol/L)	39.6 (41.7)	38.5 (37.3)	0.47	− 2.8 (− 10.6 to 5.6)
* C*_min_ (nmol/L)	31.9 (39.8)	32.2 (34.1)	0.77	1.2 (− 7.3 to 10.5)
**Tamoxifen**				
AUC_0–24h_ (nmol·h/L)	6867 (26.1)	7150 (22.9)	0.44	4.1 (− 6.6 to 16.1)
* C*_max_ (nmol/L)	401.5 (28.1)	392.6 (25.1)	0.64	− 2.2 (− 11.8 to 8.4)
* C*_min_ (nmol/mL)	257.1 (35.6)	273.0 (24.4)	0.34	6.2 (− 6.8 to 20.9)

*PK* pharmacokinetic, *CI* confidence interval, *AUC* area under the plasma-concentration–time curve, *C*_*max*_ maximum observed concentration, *C*_*min*_ minimum observed concentration

^a^Values are geometric mean (% coefficient of variation)

The plasma pharmacokinetic parameters of tamoxifen showed a clear resemblance in AUC_0–24h_ with and without green tea (RD 4.1%, 95% CI − 6.6 to 16.1%; *p* = 0.44). Likewise, the determined relative difference of tamoxifen *C*_max_ (RD − 2.2%, 95% CI − 11.8 to 8.4%; *p* = 0.64) and *C*_trough_ (RD 6.2%, 95% CI − 6.8 to 20.9%; *p* = 0.34) also shared similar results between both treatments. No differences between CYP2D6 phenotype groups and endoxifen exposure were found.

### Treatment-related adverse events

An overview of treatment-related adverse events is presented in Table 3. Headache, gastro-intestinal side-effects (e.g*.,* constipation and dyspepsia) and polyuria were reported more often during the treatment with green tea vs tamoxifen monotherapy. A few changes in liver biochemical parameters (AST, ALT, GGT) occurred during administration with green tea, as well as a creatinine increase and platelet count decrease. Hot flashes were the most reported side-effects, but its occurrence count remained the same independent of green tea consumption. Adverse events were mild and serious adverse events (grade 3 or higher) were not observed during the study period.

**Table 3:** Treatment-related adverse events, graded according to CTCAEv.5

Adverse event	Tamoxifen monotherapy (*N*)	Tamoxifen with green tea (*N*)
**Grade 1**		
** General**		
Abdominal pain	2	
Headache	2	4
Hot flashes	5	5
Restlessness		1
** Gastro-intestinal**		
Nausea	1	
Dyspepsia		1
Gastroesophageal reflux		1
Constipation		1
Belching		1
Bloating		1
** Urogenital**		
Polyuria		3
Irregular menstruation		1
Menorrhagia	1	1
** Biochemistry**		
ASAT increased		1
ALAT increased		1
GGT increased		1
Creatinine increased		1
Platelet count decreased		2
**Grade ≥ 3**	0	0

*ASAT* aspartate aminotransferase, *ALAT* alanine aminotransferase, *GGT* gamma-glutamyltransferase

## Discussion

This randomized, cross-over, pharmacokinetic study clearly demonstrated that green tea supplements did not cause a pharmacokinetic interaction with tamoxifen or endoxifen in breast cancer patients. Therefore, we can conclude that tamoxifen absorption and metabolism were not affected by green tea from a pharmacokinetic point of view. Furthermore, serious or severe green tea related adverse events were not reported during the whole study period.

These results were unexpected as preclinical studies showed that green tea did modify important targets of tamoxifen metabolism (e.g., OATP, P-glycoprotein, UGT and CYP enzymes) [23, 25–27, 34]. Several mechanisms for drug interactions resulting in an altered bioavailability or metabolism have been reported, including inhibition of influx- or efflux-transporters and cytochrome P450 enzymes [18–22]. Furthermore, other green tea–drug combinations were previously studied in humans, and significant herb–drug interactions with clinical implications were found [18, 20]. Consequently, it was hypothesized that green tea would induce changes in the systemic exposure of tamoxifen and endoxifen, but no differences in endoxifen and tamoxifen exposure between the phase with and without green tea were found in this study.

The non-significant effect is not consistent with the outcomes of a study that reported EGCG (range 3 to 10 mg/kg) significantly altered the pharmacokinetic parameters of tamoxifen in rats [35]. This animal study suggested that EGCG might be effective to obstruct CYP3A4-mediated metabolism and P-glycoprotein mediated efflux pathways in the intestine and liver. However, a lower dose EGCG (0.5 mg/kg) did not significantly alter the metabolite formation of tamoxifen in rats [35]. This phenomenon suggests a dose-dependent effect of EGCG on the pharmacokinetic profile of tamoxifen. In this trial, the EGCG dose used is equivalent to a dose of approximately 4 mg/kg.

In this study a commercially available green tea extract was administered, in what is considered a high, but safe dose for humans (2000 mg green tea per day of which 300 mg is EGCG) and in line with dosages used in previous clinical studies and with what we observe in breast cancer patients in our out-patient clinic [10, 35–39]. This EGCG dose is equivalent to approximately about 5 to 6 cups of green tea. According to the European Food and Safety Association (European agency funded by the European Union) 300 mg EGCG is comparable to the maximum mean daily EGCG intake from the consumption of regular green tea in beverage form [38]. However, it is worth noting that routes of administration other than green tea supplements (e.g., green tea beverages) may in theory affect green tea absorption and bioavailability and therefore may affect tamoxifen pharmacokinetics. Therefore, it is possible that green tea beverages show a different bioavailability of EGCG compared with green tea capsules. However, a possible interaction with the green tea beverage is less likely since similar EGCG levels are likely to be obtained in human plasma. Apparently, administration of green tea capsules influences the phase II metabolism of tamoxifen to a very limited extend.

The main reported adverse events in this trial were headaches, hot flashes, gastro-intestinal toxicity, polyuria and minor abnormalities in liver biochemical parameters. The incidences of headache, polyuria, gastro-intestinal adverse events and minor liver biochemical disturbances were increased in the green tea phase, whereas abdominal pain was more present without green tea. All reported adverse events during this study were mild (grade 1). Previous studies found similar gastro-intestinal and hepatic adverse events related to the administration of high doses of green tea [36, 37, 40]. In addition, headache, polyuria and restlessness are well-known side-effects of caffeine, one of the substituents of green tea supplements (140 mg/day, equivalent to approximately 200 mL of filtered coffee). These green tea related adverse events suggest that green tea was sufficiently absorbed, which is important because of its low oral bioavailability [13, 41, 42]. To ensure adequate green tea absorption, we administered the daily dose in two dosages and patients with known impaired drug absorption were excluded.

In conclusion, this study clearly indicated that tamoxifen and endoxifen pharmacokinetics were not affected by green tea supplements. Concomitant treatment with green tea and tamoxifen was well-tolerated in this real-life breast cancer cohort. Therefore, the use of green tea among breast cancer patients does not have to be actively discouraged by physicians.
